# Grisel's Syndrome After COVID-19 in a Pediatric Patient: A Case Report

**DOI:** 10.7759/cureus.62028

**Published:** 2024-06-09

**Authors:** Kazuhiko Hashimoto, Shunji Nishimura, Yu Shinyashiki, Koji Goto

**Affiliations:** 1 Orthopedic Surgery, Kindai University Hospital, Osakasayama, JPN

**Keywords:** non-steroidal anti-inflammatory drugs, pediatric, covid-19, female, atlantoaxial rotatory fixation

## Abstract

An eight-year-old female presenting with posterior neck pain and torticollis who had been diagnosed with coronavirus disease 2019 (COVID-19) three weeks earlier was radiographed and diagnosed with atlantoaxial rotatory fixation (AARF). Following treatment with non-steroidal anti-inflammatory drugs (NSAIDs), the posterior neck pain improved, and the torticollis was cured. Symptoms returned after two weeks, and computed tomography showed a 3.94 mm atlantodental interval and axis rotation. The patient was diagnosed with AARF relapse; symptoms resolved spontaneously prior to subsequent examination, and no further relapses were observed.

This case highlights the need for clinicians to be aware that AARF may develop after COVID-19. Treatment options should be carefully considered.

## Introduction

Atlantoaxial rotatory fixation (AARF) is an acquired fixed rotation abnormality of the C1 vertebra on C2 that interferes with neck rotation. AARF typically manifests as torticollis in children [1-3]. Moreover, AARF is a common cause of cervical pain and limited movement in children [3,4]. The clinical diagnosis of AARF is based on a fixed rotational deformity of the neck, often with the typical “Cock-Robin” position due to cervical lateral flexion, forward flexion, and rotation [5]. The diagnosis of AARF is often delayed [1-3]. AARF may be triggered by minor trauma, upper respiratory tract infection, or surgery of the oral cavity or pharynx [5-7]. Grisel's syndrome is an acquired torticollis that involves subluxation of the atlantoaxial joint (C1/2) due to inflammatory ligamentous laxity following an infectious process in the head and neck [8]. Also, it primarily affects children (68% <12 years) [8]. Herein, we describe a case of AARF that developed after severe acute respiratory syndrome coronavirus 2 (SARS-CoV-2) infection.

## Case presentation

The patient was an eight-year-old girl. The chief complaints were neck pain and inability to move the neck. The patient was diagnosed with coronavirus disease 2019 (COVID-19) three weeks prior to visiting our department, presenting with posterior neck pain, sore throat, and torticollis.

**Figure 1:**
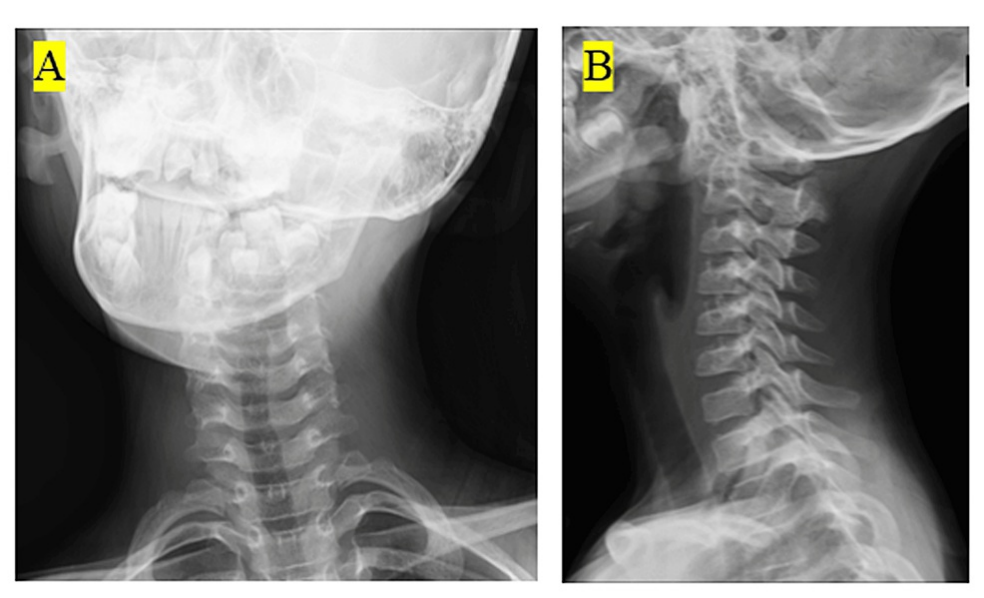
X-ray image of the cervical spine at the first visit (A) Frontal X-ray image of the cervical spine. An oblique neck position was observed. (B) Lateral radiograph of the cervical spine. Lateral alignment of the cervical spine was intact.

She was radiographed and prescribed a non-steroidal anti-inflammatory drug (1200 mg/day of acetaminophen) with a diagnosis of AARF. She refused to wear a cervical collar. Subsequently, the posterior neck pain improved three days after taking the medication internally, and the torticollis was no longer apparent. However, two weeks later, her neck symptoms flared without taking medication, and she returned to our department. The external finding was the torticollis. She also had pain in the posterior neck. Normal neurology findings were observed in the upper or lower extremities. At the time of the patient's primary visit to our department, torticollis was observed when a neck X-ray was done (Figure 1A). Lateral radiographs showed normal cervical spine alignment (Figure 1B).

**Figure 2:**
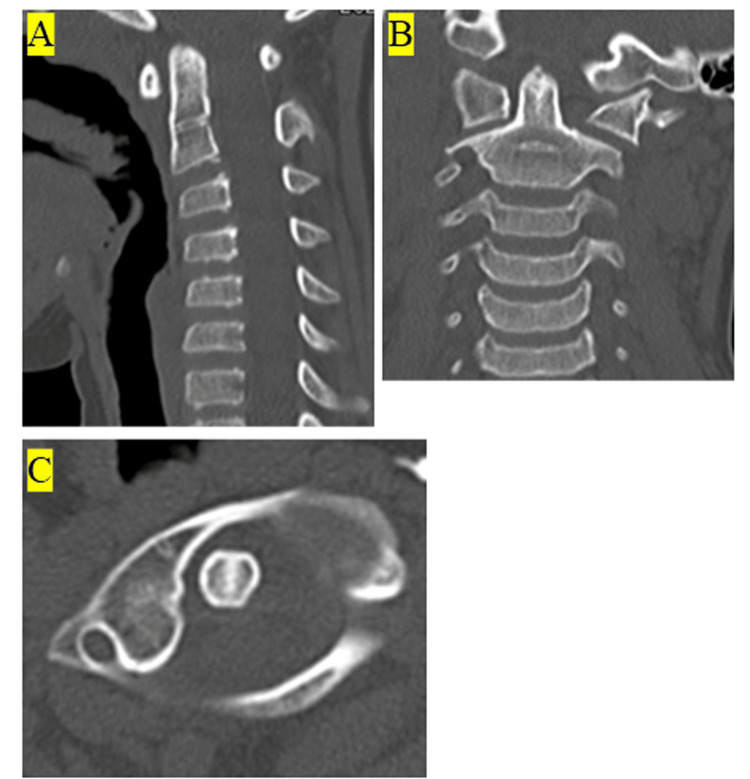
Computed tomography in the first visit (A) Computed tomography (CT) image of the lateral cervical spine. (B) CT image of the frontal cervical spine. (C) Transverse CT image of the annular and axis vertebrae. AARF was observed. AARF: Atlantoaxial rotatory fixation

Computed tomography (CT) at the second visit showed an atlantodental interval (ADI: the horizontal distance between the posterior cortex of the anterior arch of the atlas (C1) and the anterior cortex of the dens in the median (midsagittal) plane) of 3.94 mm and rotation of the axis (Figures 2A-2C).

The patient was diagnosed with AARF relapse (Fielding classification: type II) [9] and referred to a specialized hospital one day later. However, at the time of the initial visit to the referral hospital, the patient's symptoms had already been cured. Thereafter, no relapses were observed after 23 months of follow-up. Written informed consent was obtained from the patient and her mother.

## Discussion

AARF may develop after minor injuries and present as a painless neck deformity [10]. In the present report, we describe a case of AARF that developed after SARS-CoV-2 infection.

The main symptoms of AARF are cervical rotation limitation, motion pain, and stridor, which are most common in children [11]. The resultant wide spinal anteroposterior diameter rarely results in spinal cord paralysis [12].

In the current case, as in previous reports, the main complaints were posterior neck pain and stridor, with no neurological symptoms in the extremities. Asymmetric and rotational subluxation of the axis vertebrae is caused by trauma or inflammation of the pharynx and tonsils [11]. Pediatric AARF associated with non-traumatic inflammation is referred to as Grisel's syndrome [13].

Inflammation causing hyperemia associated with head and neck infections or surgical procedures may result in laxity of the periaqueductal ligaments, including the transverse and axial ligaments [14]. In addition, children have greater articulation between the interior arch of C1 and the dens and meniscus-like synovial folds at the occiput/C1 and C1/2 joints than those of adults. This has been reported as a cause of AARF [15]. Meniscus-like synovial folds can cause impingement and synovitis, leading to ligamentous laxity.

Pharyngitis (28.6%), along with cough (48%) and fever (47%), was reported as the main symptom of COVID-19 in pediatric patients [16]. In the present case, pharyngitis due to COVID-19 caused laxity of the ligaments around the atlantoaxial joints, including the transverse and auricular ligaments, which was thought to have caused AARF.

In AARF, lateral X-ray images show enlargement of the ADI and retropharyngeal space [17,18]. An ADI within 3.5 mm is considered normal in children [19]. In AARF, the ADI increases to 5.0-7.0 mm or more, resulting in subluxation of the rotated position [20]. Dens also deviate from the left or right side, as seen in CT scans [21]. In this case, the ADI showed a slight deviation of approximately 4 mm to the left.

In general, fixed classification is used as the AARF classification method [22]. Type 1 is classified as rotational fixation without anterior dislocation of the axis; Type 2, anterior dislocation of the axis of ≤ 5 mm; Type 3, anterior dislocation of the axis of ≥ 5 mm; and Type 4, posterior dislocation of the axis. This case was classified as Type 2.

Recently, there have been reports on management strategies for AARF; however, no consensus has been reached [22]. With early diagnosis and consolidation, most patients with acute AARF can be cured completely with conservative therapy, e.g., analgesics, neck collars, and traction [6]. Additionally, children are relatively easy to treat [14]. Traction therapy or surgical internal fixation is necessary for severe cases that are difficult to realign, prone to redislocation, or remain unstable after realignment [6,22]. This case was relatively mild, and it was considered to have spontaneously cleared.

It may be not uncommon for pharyngitis caused by COVID-19 to result in AARF. However, patients with COVID-19 often visit an internist first, and we believe that it is highly likely that AARF will be missed at the time of these initial visits. Therefore, we believe that it is notable that an oblique neck in a patient with COVID-19 may potentially be a case of AARF as evidenced by this case report.

## Conclusions

In conclusion, we report a case of AARF that developed after COVID-19. These findings indicate that clinicians should be aware that AARF can develop after respiratory infections like COVID-19. In addition, careful consideration should be given to whether AARF should be treated surgically or conservatively.
